# Vaccine Coverage among Children with and without Intellectual Disabilities in the UK: Cross Sectional Study

**DOI:** 10.1186/s12889-019-7106-5

**Published:** 2019-06-13

**Authors:** Eric Emerson, Janet Robertson, Susannah Baines, Chris Hatton

**Affiliations:** 1 Centre for Disability Research, Lancaster University, Lancaster, LA1 4YT UK; 2 Centre for Disability Research and Policy, Faculty of Health Sciences, University of Sydney, Sydney, NSW 2006 Australia

**Keywords:** Intellectual disability, Children, Vaccination

## Abstract

**Background:**

Universal childhood vaccination programmes form a core component of child health policies in most countries, including the UK. Achieving high coverage rates of vaccines is critical for establishing ‘herd immunity’ and preventing disease outbreaks. Evidence from the UK has identified several groups of children who are at risk of not being fully immunised. Our aim was to determine whether children with intellectual disabilities constitute one such group.

**Methods:**

Secondary analysis of parental report data on child vaccination collected in the UK’s Millennium Cohort Study when the children were 9 months, 3 years, 5 years and 14 years old.

**Results:**

With one exception (MMR coverage at age 5) vaccination coverage rates were lower for children with intellectual disabilities (when compared to children without intellectual disability) for all vaccinations at all ages. Complete coverage rates were significantly lower for children with intellectual disabilities at ages 9 months (unadjusted PRR non-vaccination = 2.03 (1.14–3.60), *p* < 0.05) and 3 years (unadjusted PRR = 2.16 (1.06–4.43), *p* < 0.05), but not at age 5 years (unadjusted PRR = 1.91 (0.67–5.49)). HPV vaccination was lower (but not significantly so) at age 14 (PRR = 1.83 (0.99–3.37), *p* = 0.054). Adjusting PRRs for between group differences in family socio-economic position and other factors associated with coverage reduced the strength of association between intellectual disability and coverage at all ages. However, incomplete vaccination remained significantly elevated for children with intellectual disabilities at ages 9 months and 3 years. There were no statistically significant differences between parents of children with/without intellectual disability regarding the reasons given for non-vaccination.

**Conclusions:**

Children with intellectual disabilities in the UK are at increased risk of vaccine preventable diseases. This may jeopardise their own health, the health of younger siblings and may also compromise herd immunity.

## Background

Universal childhood vaccination programmes have been described as ‘one of the most successful and cost-effective health interventions known’ [1] and form a core component of child health policies in most countries, including the UK [2, 3]. Achieving high coverage rates of vaccines is critical for establishing ‘herd immunity’ and preventing disease outbreaks.

Evidence from the UK has identified a number of groups of children who are at risk of not being fully immunised including children: living in more deprived areas; of teenage or lone parents; of unemployed parents; not registered with a GP; with older siblings; from some minority ethnic groups; from non-English speaking families; whose families are travellers, asylum seekers or are homeless [4–10]. Identifying and tailoring interventions to the needs of such ‘at risk’ groups will be critical to achieving high coverage rates.

A present it is not known whether, in the UK, children with intellectual disabilities constitute one such group. Only two small scale studies have addressed this issue in the UK [11, 12], although some evidence of lower coverage among children with intellectual disability from population based studies is available from Taiwan [13, 14] and Australia [15]. Tuffrey and Finlay [12] report lower vaccination rates for pertussis, measles and rubella for children with intellectual or physical disabilities attending special schools in one health district in England. More recently, MacLeod and Tuffrey [11] have reported lower rates of human papillomavirus vaccination among girls attending schools for children with intellectual disability in one health district in England.

Two sources of evidence suggest that it is likely that vaccination coverage among children with intellectual disabilities may be lower than the national average. First, indicators of lower socio-economic position have been associated in the UK with lower childhood vaccination coverage rates (see above) and an increased prevalence of intellectual disability [16]. Second, there is some limited evidence that historically in the UK paediatricians may have advised parents of children with intellectual and developmental disabilities not to immunise [17].

The primary aim of our paper is to determine whether, in the UK, children with intellectual disabilities are at risk of non-vaccination. A secondary aim is to determine the extent to which any differences in coverage rates between children with and without intellectual disability may be accounted for by between-group differences in family socio-economic position and/or exposure to other established risk factors for low coverage.

## Methods

We analysed data collected in Waves 1–3 and 6 of the UK’s Millennium Cohort Study (MCS). The fourth in the series of British birth cohort studies, MCS is designed to follow a cohort of over 18,000 children born in the UK between 2000 and 2002. MCS data are managed by the Centre for Longitudinal Studies at the University of London (www.cls.ioe.ac.uk/) and were downloaded from the UK Data Service (http://ukdataservice.ac.uk/). Full details of the design of MCS are available in published reports and technical papers [18–20].

### Sampling

Families were randomly selected from Child Benefit Records, a universal welfare benefit. Sampling was geographically clustered in 398 randomly selected electoral wards in the UK. Sampling was stratified to over-sample children from ethnic minority groups, disadvantaged communities and children born in Wales, Scotland and Northern Ireland [21]. The first survey (MCS1) took place when children were 9 months old (*n* = 18,552 families). Vaccination information was collected when the children were followed up at ages three (MCS2; 14,898 families interviewed), five (MCS3; 14,678 families interviewed) and 14 (MCS6; 11,173 families interviewed). Information was collected on all children falling within the designated birth date window.

### Procedure

The data used in the present study were collected by either parental report or direct cognitive testing of the child.

### Identification of children with intellectual disabilities

Intellectual disability was identified from the results of assessments of child cognitive ability at ages seven, five and 3 years and, for a small number of children for who no test results were available, parental report of receipt of special education services and child attainment. Full details of these procedures have been published in BMC Public Health [22] and elsewhere [23]. This procedure led to the identification of 671 of 18,552 (3.6%) children. As expected, boys were significantly more likely than girls to be identified as having intellectual disability (4.3% vs 2.6%; OR = 1.67, 95% CI 1.42–1.96).

### Vaccination uptake

The UK universal vaccination schedule relevant to the MCS cohort was as follows: (1) due at age 8–16 weeks, primary vaccines (Diphtheria, Tetanus and Pertussis (DTP); *Haemophilus influenzae* type b (Hib); Oral Polio; Meningococcal group C (Men C)); (2) due at age 1, Measles, Mumps and Rubella (MMR); (3) due at age 3 years, 4 months, preschool booster (DTP; Polio - oral or inactivated; MMR); (4) due at age 12–13 human papillomavirus (HPV).

### Uptake

Vaccination uptake was based on primary parental informant report. At all Waves informants were requested to consult parent-held child vaccination records to answer questions about vaccinations. At Wave 1 information was only recorded on whether the target child had no, some or all recommended vaccinations. At Waves 2 and 3 information was recorded on the uptake of each recommended vaccination. We used Wave 1 to 3 data to generate three binary summary variables at each Wave (fully or partially vaccinated vs not vaccinated, fully vaccinated vs not vaccinated, partially vaccinated vs not vaccinated). At Wave 6 information was only recorded on whether the target child, if female, had received HPV vaccination. Vaccination coverage data was available for 18,528 children at age 9 months (99.9% of participating children), 14,776 children at age 3 years (99.2% of participating children), 14,650 children at age 5 years (99.8% of participating children) and 5488 girls at age 14 years (98.6% of participating girls).

### Reasons given for non-uptake

At each wave (and at Waves 2 and 3 for each vaccine) parental informants were asked open ended questions about the reason for non-vaccination. Responses were coded by the MCS team into categories that varied across waves both in content and number of categories (up to 44 at Wave 3) Given the small numbers involved we grouped specific reasons into seven categories: parental choice (e.g., concerns about side effects, preference for homeopathic treatments); service/administration errors (e.g., vaccine not available); child unwell at time of vaccination; adverse reactions to previous vaccinations or health-related contra-indication; family disorganisation (e.g., not keeping or making appointment); appointment pending; other (e.g., don’t know, vague or irrelevant answer).

### Potential confounding variables

Given the strong association between immunisation uptake at age 9 months (Wave 1) and subsequent immunisation practices [7, 24], potential confounding variables were selected from Wave 1 data. Selection was based on availability in MCS data and evidence from previous studies that potential confounders were associated with immunisation uptake and may be more or less common among families of children with intellectual disability when compared to other families. We identified two broad groups of potential confounding variables; indicators of family socio-economic position and other factors associated with family composition and country of residence.

### Indicators of family socio-economic position

Previous research using the MCS has indicated that lower uptake of immunisations was associated with a number of variables indicative of family socio-economic position including residence in a disadvantaged neighbourhood, lone or teenaged parenthood, maternal smoking in pregnancy, maternal educational attainment, maternal employment status and ethnicity [6–8]. Low family socio-economic position is also associated with an increased risk of intellectual disability [16]. As a result, we included the following indicators of family socio-economic position in our analyses:

- Income poverty: operationalised as living in a household with equivalised income lower than 60% of the national median [25].
- Low household assets: operationalised as lacking two or more household assets from a list of eight common household assets (e.g., fridge, freezer, washing machine, microwave, home computer).
- Living in workless household: operationalised as no adult in the household being in paid employment.
- Maternal educational attainment: operationalised on the basis of parental informant report as degree/diploma level, GCSE grade C or above, lower than GCSE grade C or above.
- Residence in a disadvantaged neighbourhood: operationalised as living in an area in the lowest national quintile on a measure of multiple deprivation (e.g., [26]).
- Lone parenthood: operationalised as not cohabiting with another parent figure.

### Other potential confounders

Previous research using the MCS has indicated that lower uptake of immunisations was associated with a number of other variables not necessarily associated with family socio-economic position including living with siblings, younger and older mothers, minority ethnicity, child born in England (as opposed to other home countries), maternal smoking in pregnancy and child hospital admission in first 9 months of life [6–8]. While the association between most of these variables and risk of intellectual disability is unknown, we included them in our analyses (primarily as binary variables) based on parental informant report at Wave 1. The one exception to their inclusion as binary variables was maternal age which was included as a four-level ordinal variable based on population quartiles (14–23, 24–28, 29–32, 33+).

### Approach to analysis

All analyses were undertaken in Stata 10 SE using svy command to take account of the initial sampling design and biases in recruitment and retention at each Wave [27]. To avoid the statistical problems associated with the clustering of multiple births within households, the present analyses are restricted to the first named target child in multiple birth households.

First, bivariate descriptive analyses were undertaken to estimate vaccination coverage rates (with 95% confidence intervals) for children with and without intellectual disabilities. Chi Square was used to test the statistical significance of between group differences in coverage rates.

In the second stage of analysis we used Poisson regression to calculate prevalence rate ratios (PRRs) to estimate the strength and statistical significance of differences in vaccination coverage rates between children with and without intellectual disabilities [28, 29]. The base for these analyses was children without intellectual disability. The dependent variable was the probability of not being vaccinated. PRRs were estimated under three conditions: (1) simple unadjusted; (2) adjusted for potential confounding variables associated with family socio-economic position; (3) adjusted for all potential confounding variables.

All analyses used the Stata ‘syv’ commands to address the complex clustered sample design and utilised supplied sampling weights to take account of biases in initial recruitment and retention.

## Results

Vaccination coverage rates and prevalence rate ratios for non-uptake of vaccinations are presented in Table 1. For all ages and for both groups of children complete coverage rates are high (range 84.9–95.2%). However, with one exception (MMR coverage at age 5) coverage rates were lower for children with intellectual disabilities. Complete coverage rates were significantly lower for children with intellectual disabilities at ages 9 months (unadjusted PRR = 2.03 (1.14–3.60), *p* < 0.05) and 3 years (unadjusted PRR = 2.16 (1.06–4.43), *p* < 0.05), but not at age 5 years (unadjusted PRR = 1.91 (0.67–5.49)). Adjusting PRRs for between group differences in family socio-economic position and other factors associated with coverage significantly reduced the strength of association between intellectual disability and coverage at age 9 months and non-significantly reduced the strength of association between intellectual disability and coverage at ages three and 5 years.

**Table 1:** Vaccination coverage among children with and without intellectual disabilities in the UK

	% Vaccination coverage (with 95% CI)		Rate ratios for *non-uptake* among children with intellectual disability (reference group = other children)		
	Children with intellectual disabilities	Other children	Unadjusted	Model 1 (SEP)	Model 2 (SEP + other factors)
Age 9 months	(*n* = 551)	(*n* = 17,986)			
Fully or partially vaccinated	97.6% (95.7–98.6)	98.7% (98.5–99.0)	1.93 (1.09–3.43)*	1.33 (0.69–2.57)	1.33 (0.71–2.53)
Fully vaccinated	89.5% (86.2–92.1)	95.2% (94.8–95.7)	2.03 (1.14–3.60)*	1.38 (0.71–2.637)	1.38 (0.73–2.63)
Partially vaccinated	8.0% (5.8–11.0)	3.5% (3.2–3.9)	0.88 (0.53–1.47)	0.90 (0.50–1.63)	0.96 (0.54–1.70)
No vaccinations	2.4% (1.4–4.3)	1.3% (1.1–1.6)			
Age 3 years	(*n* = 521)	(*n* = 14,261)			
Polio complete	97.3% (95.1–98.6)	98.8% (98.6–99.0)	2.27 (1.20–4.29)*	2.22 (1.16–4.23)*	2.18 (1.13–4.19)*
Diptheria complete	98.2% (96.3–99.1)	98.8% (98.6–99.1)	1.59 (0.78–3.23)	1.48 (0.70–3.13)	1.47 (0.70–3.15)
Tetanus complete	97.7% (95.8–98.8)	98.9% (98.6–99.1)	2.02 (1.07–3.82)*	1.89 (0.97–3.66)	1.90 (0.97–3.71)
Pertussis complete	96.4% (93.8–98.0)	98.4% (98.1–98.7)	2.23 (1.27–3.92)**	2.07 (1.16–3.70)*	2.12 (1.19–3.79)*
Hib complete	96.5% (93.9–98.0)	98.2% (97.8–98.5)	1.95 (1.10–3.45)*	1.78 (0.98–3.23)	1.78 (0.98–3.23)
Meningitis complete	96.7% (94.2–98.1)	98.1% (97.7–98.4)	1.73 (0.98–3.07)	1.59 (0.87–2.92)	1.62 (0.87–3.01)
MMR	91.6% (88.1–94.2)	93.9% (93.2–94.6)	1.38 (0.96–1.09)	1.24 (0.85–1.80)	1.18 (0.81–1.72)
Fully or partially vaccinated	98.1% (96.2–99.0)	99.1% (98.8–99.3)	2.06 (1.01–4.22)*	1.77 (0.82–3.79)	1.78 (0.83–3.81)
Fully vaccinated	84.9% (80.8–88.3)	90.1% (89.2–90.9)	2.16 (1.06–4.43)*	1.82 (0.85–3.92)	1.83 (0.86–3.91)
Partially vaccinated	13.2% (10.1–17.1)	9.0% (8.2–9.7)	1.35 (0.70–2.63)	1.35 (0.66–2.77)	1.35 (0.66–2.75)
No vaccinations	1.9% (1.0–3.8)	0.9% (0.7–1.2)			
Age 5 years	(*n* = 524)	(n = 14,365)			
Booster DTP	94.5% (91.9–96.3)	96.0% (95.5–96.4)	1.38 (0.92–2.06)	1.19 (0.79–1.79)	1.17 (0.77–1.77)
All 3 doses combined DTP	98.7% (97.0–99.5)	99.2% (98.9–99.4)	1.56 (0.64–3.81)	1.43 (0.54–3.77)	1.36 (0.51–3.63)
Booster polio	94.9% (92.4–96.7)	95.2% (94.6–95.7)	1.06 (0.69–1.61)	0.99 (0.64–1.94)	0.98 (0.63–1.54)
All three doses Polio	98.7% (97.1–99.5)	99.2% (99.0–99.4)	1.61 (0.68–3.79)	1.57 (0.62–4.01)	1.51 (0.60–3.84)
All 3 doses Meningitis C	97.8% (95.5–98.9)	98.4% (98.1–98.7)	1.40 (0.70–2.82)	1.33 (0.63–2.79)	1.32 (0.63–2.74)
MMR	96.9% (95.0–98.1)	96.8% (96.2–97.2)	0.96 (0.58–1.58)	1.07 (0.64–1.79)	1.01 (0.60–1.70)
Fully or partially vaccinated	99.1% (97.4–99.7)	99.5% (99.3–99.6)	1.88 (0.66–5.39)	2.01 (0.61–6.59)	1.93 (0.59–6.35)
Fully vaccinated	87.4% (83.9–90.2)	89.3% (88.4–90.1)	1.91 (0.67–5.49)	1.97 (0.60–6.44)	1.88 (0.58–6.13)
Partially vaccinated	11.7% (9.1–14.9)	10.2% (9.5–11.1)	1.60 (0.59–4.36)	2.18 (0.69–6.88)	2.20 (0.72–6.77)
No vaccinations	1.0% (0.3–2.7)	0.5% (0.4–0.7)			
Age 14 years	(*n* = 149)	(*n* = 4938)			
HPV (girls only)	87.4% (77.9–93.2)	93.1% (92.1–94.0)	1.83 (0.99–3.37)	1.59 (0.83–3.03)	1.52 (0.78–2.98)

Note: * *p* < 0.05, ** *p* < 0.01, *** *p* < 0.001

*N* = weighted sample size

Reasons given by informants for non-vaccination are presented in Table 2. There were few statistically significant between group differences in the probability of citing specific reasons for non-vaccination. The only consistent differences were that at age 9 months and 3 years reasons for non-vaccination based on parental choice were significantly more frequently cited by parents of children without intellectual disability. The high rates of parental choice reasons given at age 3 were primarily driven by low uptake of, and concerns about the safety of, MMR vaccination.

**Table 2:** Parental reasons given for non-vaccination

Reason	Child age	Children with intellectual disabilities (% with 95% CI)	Other children (% with 95% CI)	Adjusted F
Parental choice	9 months	1.3% (0.3–4.5)	10.2% (7.3–14.0)	14.85***
	3 years	62.5% (47.3–75.5)	76.8% (73.9–79.5)	4.40*
	5 years	16.0% (7.2–31.9)	14.8% (12.4–17.5)	0.04
	14 years	46.7% (20.1–75.4)	42.0% (35.7–48.6)	0.09
Service/administration errors	9 months	8.0% (1.9–27.7)	9.1% (6.5–12.5)	0.03
	3 years	5.1% (1.3–17.5)	3.6% (2.7–4.7)	0.27
	5 years	30.9% (18.3–47.2)	26.1% (23.1–29.3)	0.42
	14 years	14.7% (3.5–45.4)	12.8% (9.2–17.6)	0.04
Child unwell at time of planned vaccination	9 months	8.6% (3.1–21.4)	39.9% (35.1–44.8)	0.53
	3 years	8.6% (3.1–21.4)	6.3% (5.1–7.9)	0.36
	5 years	3.3% (1.1–9.4)	5.6% (4.1–7.7)	0.93
	14 years	13.5% (2.0–54.7)	9.3% (5.8–14.5)	0.15
Adverse reactions or health-related contra-indication	9 months	5.9% (1.5–20.6)	9.1% (6.6–12.4)	0.39
	3 years	8.7% (3.3–21.2)	9.3% (7.8–11.0)	0.02
	5 years	20.1% (9.9–36.5)	5.7 (4.4–7.5)	12.97***
	14 years	0.0% (0.0–16.8)	1.6% (0.6–4.3)	0.21
Family disorganisation	9 months	8.9% (2.7–26.0)	14.5% (11.3–18.4)	0.70
	3 years	3.6% (0.5–21.0)	2.9% (2.1–4.1)	0.04
	5 years	0.0% (0.0–8.0)	5.7% (4.4–7.3)	1.97
	14 years	0.0% (0.0–16.8)	11.0% (7.5–15.8)	1.42
Appointment pending	9 months	25.8% (13.2–44.4)	20.8% (17.0–25.2)	0.39
	3 years	1.8% (0.5–6.4)	3.1% (2.2–4.3)	0.65
	5 years	21.8% (10.6–39.7)	30.0% (26.8–37.5)	0.95
	14 years	Information not collected		
Other	9 months	15.4% (6.5–32.5)	12.0% (9.1–15.7)	0.32
	3 years	20.8% (11.6–34.6)	12.0% (10.1–14.2)	3.52
	5 years	14.9% (6.6–30.2)	24.4% (21.3–27.9)	1.83
	14 years	25.1% (7.8–56.9)	26.4% (20.7–32.9)	0.01

Note: Base for % is the number of parents giving one or more reason at each wave

* *p* < 0.05, **** *p* < 0.001

## Discussion

This is the first study to report on early childhood vaccination coverage among a nationally representative sample of UK children with and without intellectual disability. The main findings are: (1) at ages 9 months, three and 5 years complete coverage rates are reasonably high for both groups of children; (2) with one exception (MMR coverage at age 5) coverage rates were lower for children with intellectual disabilities for all vaccinations (when compared to children without intellectual disability); (3) complete coverage rates were significantly lower for children with intellectual disabilities at ages 9 months and 3 years, and lower (but not significantly so) at age 5 years; (4) while adjusting PRRs for between group differences in family socio-economic position and other factors associated with coverage reduced the strength of association between intellectual disability and coverage at all ages, incomplete vaccination remained significantly elevated for children with intellectual disabilities at ages 9 months and 3 years; (5) there were no statistically significant differences between parents of children with/without intellectual disability regarding the broad classes of reasons given for non-vaccination. These results are broadly consistent with evidence of lower coverage among children with intellectual disability from population based studies undertaken in Taiwan [13, 14] and Australia [15].

The results suggest that a significant proportion of the risk of non-uptake among children with intellectual disability is plausibly related to the association between intellectual disability and lower family socio-economic position. However, risk of non-vaccination among children with intellectual disability remained elevated when controlling for these between-group, differences. Further research including mixed methods and qualitative studies is needed to identify the reasons for non-vaccination among children with intellectual disabilities, especially in early childhood prior to school entry. At 9 months of age, less than 5% of parents of children with intellectual disability reported that non-vaccination was a matter of parental choice, while 65% reported other reasons including apparent service failures (e.g., cancelled appointment), child illness at the time of appointment, missed appointments and parents being unaware that vaccination was recommended. While dominance of other reasons was reversed at age three (with just under 50% of parents of children with intellectual disability reported that non-vaccination was a matter of parental choice), it needs to be kept in mind that these data were primarily collected in 2003 at the peak of the controversy of the association between the combined MMR vaccine and autism [6].

The results also suggested that differences in primary vaccination coverage are minimal at age 5 (an age at which all children will have entered school). While children with intellectual disabilities do appear to catch up with their peers with regards to vaccination coverage, it should be noted that children receiving vaccinations late remain susceptible to vaccine preventable diseases which may jeopardise their own health, the health of younger siblings and may also compromise herd immunity increasing the risk of disease outbreaks [30].

The primary strength of the present study lies in its use of a sizable cohort of children representative of the population of children growing up in the UK at the beginning of the new millennium. However, as in all studies, there were limitations that impact the interpretation of these findings. First, the MCS utilised abbreviated scales of cognitive functioning (rather than complete IQ tests). While it is common practice in such instances to derive a proxy measure of IQ (cf., [22, 31, 32, 33]), the association between the proxy and full measure is unknown. As a result, it is possible that there was some misclassification of intellectual disability status. Second, while the overall sample was relatively large, it was of insufficient size to disaggregate results for the intellectual disability group by such potentially important factors as gender, ethnicity and severity of intellectual disability. Finally, while evidence in general suggests that parental report may be an unreliable measure of vaccination [34], recent research using the MCS and linked data in Wales has reported high concordance between parental reported and child health recorded MMR status [30].

## Conclusions

Children with intellectual disabilities in the UK are at increased risk of vaccine preventable diseases. This may jeopardise their own health, the health of younger siblings and may also compromise herd immunity.

## Data Availability

The datasets analysed during the current study are available to researchers on application through the UK Data Service repository (https://discover.ukdataservice.ac.uk). We received administrative permission to access and use these data.

## Abbreviations

BAS: British Ability Scales
DTP: Diphtheria, Tetanus and Pertussis
GCSE: General Certificate of Secondary Education
Hib: *Haemophilus influenzae* type b
HPV: Human papillomavirus
IQ: Intelligence quotient
MCS: Millennium Cohort Study
Men C: Meningococcal group C
MMR: Measles, Mumps and Rubella
NFER: National Foundation for Educational Research
PRR: Prevalence rate ratio
